# Climate change impact on fluvial flooding in the Indian sub-basin: A case study on the Adyar sub-basin

**DOI:** 10.1371/journal.pone.0216461

**Published:** 2019-05-14

**Authors:** Andimuthu Ramachandran, Kandasamy Palanivelu, B. V. Mudgal, Anushiya Jeganathan, Sankar Guganesh, Balu Abinaya, Arunbabu Elangovan

**Affiliations:** 1 Centre for Climate Change and Adaptation Research, Anna University, Chennai, Tamil Nadu, India; 2 Centre for Water Resources, Anna University, Chennai, Tamil Nadu, India; Universidade de Aveiro, PORTUGAL

## Abstract

Flooding is one of the most disastrous global hazards, which has been occurring more frequently in recent times. It is observed that climate change is likely to increase the intensity and the frequency of floods and river basins have become more vulnerable to fluvial flooding. In this study, the impact of climate change on fluvial flooding was analyzed over the Adyar sub-basin. This study applied statistically downscaled Global Climate Model (GCM) data in a CMIP5 dataset of IPCC Assessment Report 5 (AR5). Based on the performance to simulate the observed climate, four GCMs, namely, cesm1-cam5, mpi-esm-mr, ncar-ccsm4, and bnu-esm, for RCP 4.5 were selected for projections of the future scenario. The Intensity-Duration-Frequency (IDF) curves for the past and future scenarios were derived from the IMD-observed and GCM-projected rainfall data. Integrated flood modeling was performed with hydrologic (HEC-HMS) and hydraulic (HEC-RAS) models. Finally, in order to visualize the inundation areas according to the future climate projection, flood inundation maps were prepared geospatially using the ArcGIS software. For the 100-year return period, the results predict that the peak discharge for the future climate scenario would increase by 34.3%–91.9% as compared to the present climate scenario. Similarly, the future projections show an increase in the flooded area ranging from 12.6% to 26.4% based on GCMs. This simulation helps in understanding the flood risk over the Adyar sub-basin under the changing climate and the requirement for the regulation of developmental activities over the flood-prone areas.

## Introduction

Nowadays, the global climate is changing significantly [1]. These changes lead to an increase in the global average temperature and variations in the rainfall intensity and frequency. Consequently, increase in the rainfall intensity and frequency will increase the runoff and peak discharge leading to an increase in flood events [2–5]. Flooding is considered one of the most disastrous global hazards which has been occurring more frequently in recent times [6–9]. Due to climate change, there could be a possible increase in flood hazards in the future [10]. Under climate change, developing countries like India are more vulnerable to flooding, which causes considerable economic losses [11,12]. Due to the increase in extreme rainfall events, the impacts of climate change on floods severely affect the Indian river basins located in low-lying floodplains [13,14]. However, these impacts vary from place to place on a regional scale under climate change [15,16]. For sustainable development, a basin-scale assessment of impacts of climate change on flooding is necessary [17,18]. The impact studies on the affected basins under climate change were started off as a result of the recent flooding events across the country [12,19,20].

To project more precise climatic conditions, the recent climate impact studies have used Global Climate Models (GCMs) from the Coupled Model Intercomparison Project Phase 5 (CMIP5) dataset for Assessment Report 5 (AR5) of the Intergovernmental Panel on Climate Change (IPCC) [21]. In order to predict the flood risk due to climate change, these GCMs can be applied to project the future climate [6]. The projections of the future climate using GCMs are at a global scale but they could be downscaled to a basin or a sub-basin level [12]. To assess the possible impacts of climate change, various studies have applied the widely used statistically downscaled climate projections [12,21]. For the estimation of floods, this GCM dataset can be statistically downscaled to a sub-basin level.

Generally, the estimation of floods is carried out by incorporating meteorological and hydrological data by using Intensity-Duration-Frequency (IDF) curves in the hydrologic model [22]. In order to simulate the extreme rainfall intensity for a river basin in hydrological modelling, IDF curves are used [23]. IDF curves are derived by using rainfall time series data to reproduce the intensity and frequency of rainfall for different durations [24]. A critical rainfall depth for different return periods is calculated from the IDF curves and distributed throughout the river basin [25]. Flood modelling requires both the hydrologic and hydraulic models. A hydrologic model is used to simulate flood discharges, whereas the hydraulic model simulates inundation levels of floods [26]. Hydrologic modelling can deal with the problems related to flooding at a basin or a sub-basin scale. HEC-HMS is the most commonly used hydrologic model, which is used in many river basin studies [12,17,27–29]. For hydraulic modelling, HEC-RAS is used for the simulation of floods for impact assessment studies [7,17,18,30–32]. Various studies successfully integrated both the HEC-HMS and HEC-RAS models at a regional scale [17,27,30]. This approach of integrating HEC-HMS with HEC-RAS is considered as an efficient tool for flood inundation because GIS is an integral part of this approach [26].

Surya and Mudgal [17] analyzed the impact of land use change on flooding over the Adyar sub-basin by integrating both the hydrologic and hydraulic models, but the impact of climate change was not considered in the analysis. Anushiya and Ramachandran [33] studied the changes in water balance components of the Chennai basin under present and future climate scenarios, but flood assessment was not addressed in this study. Though the Adyar sub-basin is receiving more floods in recent times, the climate change analysis was not used in the flood assessment studies carried out so far [17,34]. The Adyar sub-basin experienced severe flooding during November-December 2015 which is approximately a 165-year flood event. The severity of the flood was very extreme, indicating the need for the assessment of flooding under climate change scenarios.

Based on the observed rainfall and the future GCM projections, this study addresses the impacts of climate change on fluvial flooding with an integrated analysis of the hydrologic and hydraulic models in order to develop flood inundation maps over the Adyar sub-basin for the present and future climate change scenario. Flood inundation mapping is used to identify the possible flooding areas for different return periods. The simulations of flood depths and areas will help the planners to distinguish floodplains and regulate the development in hazardous areas.

## Materials and methods

### Study area

**Fig 1:**
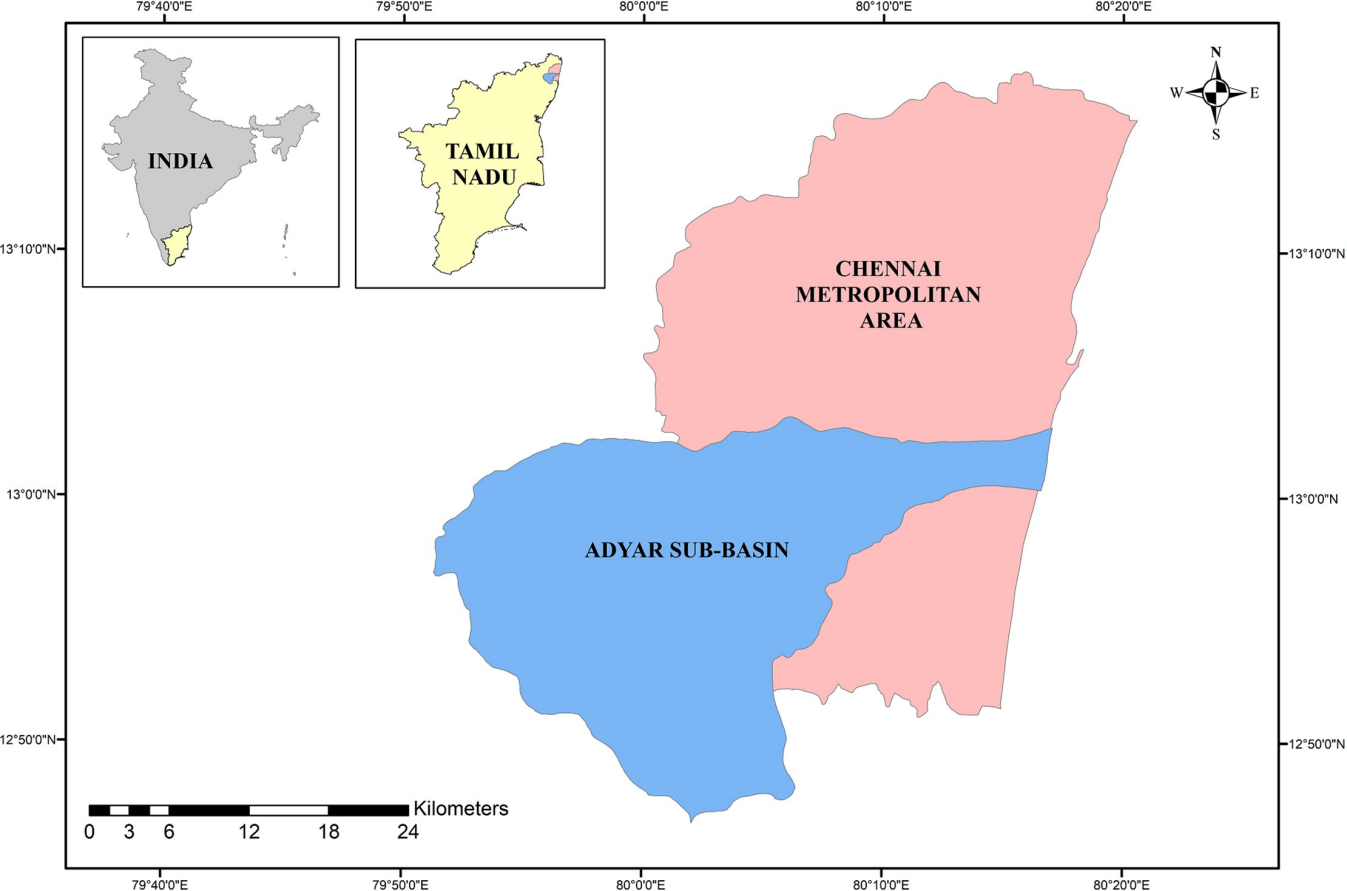
Study area.

The Adyar sub-basin, located between 13°3′19″N, 79°51ʹ12″E and 12°46ʹ37″N, 80°17'6"E is a low-lying area in the northeastern coast of Tamil Nadu, India (Fig 1).

The Adyar river originates from a group of tanks present in the Kancheepuram district. Though it starts from a group of tanks, it appears as a river only after receiving the surplus from the Chembarambakam tank located within the sub-basin. The length of the Adyar river is 42.5 km, and from the downstream of the river, 15 km of this length comes under the Chennai city limits. On the whole, the width of the Adyar river changes from 20 m to 200 m. The total catchment area of the Adyar sub-basin is around 700 km^2^, and 24% of this area is urbanized. The Adyar sub-basin is considered as one of the rapidly urbanizing areas since 2000. The Adyar sub-basin is covered by the alluvium soil in major parts, coastal sand prevails in the downstream area of the river basin, and sand and silt cover the middle and upper parts of the river basin [35].

During the period 1975–2015, the average annual rainfall over the Adyar sub-basin has been observed to be 1472 mm. The contribution of different seasons to the annual rainfall is as follows: post monsoon (October to December) rainfall is the major contributor (59.6%) followed by monsoon (June to September) rainfall (32.6%), summer (March to May) rainfall (4.9%), and finally winter (January to February) rainfall (2.8%). The Adyar sub-basin is a flat terrain with slight undulations, and hence the Adyar river is almost stagnant except during rainy seasons. Any unexpected rainfall over the sub-basin rapidly increases the water level in the river due to the flat terrain of the Adyar sub-basin. Hence, it increases the risk of flooding in the Adyar river and its surrounding areas [17].

Many flood events have occurred on the Adyar river in the past claiming many lives, properties, and infrastructure systems [17]. During 1976, 1985, 2005, 2008, and 2015, the Adyar river has inundated its banks and caused extensive flood damages (Table 1).

**Table 1:** History of flooding on the Adyar river.

Year	Experience	Flood discharge
1976	Adyar river overflowed and inundated nearby houses; Surplus water could not enter into the sea due to high tide (346.6 mm/day rainfall was recorded)	792.96 cumecs (28,000 cusecs)
1985	Submergence of encroachments near the banks of Adyar river (729 mm rainfall was recorded in three days)	1784.16 cumecs (63,000 cusecs)
1996	Surplus water from Chembarambakkam tank mixed into Adyar river (282.2 mm/day rainfall was recorded)	566.4 cumecs (20,000 cusecs).
2005	Adyar river was flooded (361 mm rainfall was recorded in two days)	1132.8 cumecs (40,000 cusecs)
2008	Flood in Adyar river. Backwater due to high tide (322 mm rainfall was recorded in two days)	424.8 cumecs (15,000 cusecs)
2015	Severe rainfall over the catchments of the Adyar river. Surplus water from Chembarambakkam tank mixed into Adyar river (345.1 mm/day rainfall was recorded)	~3000 cumecs (~105000 cusecs)

(Source: http://www.cmdachennai.gov.in/pdfs/SeminarOnWaterways/5.pdf)

In the past, frequent flooding has caused loss of property, affected the lives of many slum dwellers through displacement and heavy expenditure on their relief and rehabilitation, incurred loss to industry and business, and damaged the infrastructure. The flood occurred in December 2015 had a serious impact on the domestic, industrial, and commercial sectors in and around the Adyar sub-basin. It was reported that the areas such as Chennai International Airport, Saidapet, Kotturpuram, and Mudichur were badly affected by the flood along the Adyar river. Within a short period, this flood event caused severe socioeconomic and environmental damage. Thus, the assessment of risk due to flood becomes necessary over the Adyar sub-basin in order to increase its resilience against flooding along with climate change.

### Observed and projected rainfall

The Adyar sub-basin has one weather monitoring station, i.e., Meenambakkam station, established by the India Meteorological Department (IMD). It is the first and the oldest observatory that was established two centuries ago at Chennai in 1792. For the period 1975–2015, the daily rainfall data of the Meenambakkam station were obtained by the Regional Meteorological Centre (RMC), IMD, Chennai. For the Meenambakkam station, the seasonal and annual analyses of rainfall were performed on the basis of the observed rainfall data. According to the IMD guidelines, extremely heavy rainfall events (>244.5 mm/day) were analyzed during 1975–2015.

GCMs were used to project the future rainfall data for the period 2015–2085. In this study, raw GCM data were used in a CMIP5 dataset of IPCC AR5. Moreover, four Representative Concentration Pathways (RCPs), namely, RCP 2.6, RCP 4.5, RCP 6.0, and RCP 8.5, were used, each representing different anthropogenic radiative forces on the atmosphere [36]. Based on the observed climatic conditions, RCP 4.5 is observed to be more suitable for the Indian subcontinent [37]. A total of 26 GCMs are available for RCP 4.5 in the CCAFS website http://ccafs-climate.org/. First, these GCM data were statistically downscaled and bias-corrected by using the portal provided within the CCAFS website and then the bias-corrected GCM data were downloaded. Applying the pattern correlation (CORR), root-mean-square error (RMSE), and percentage bias, the performance of all 26 models were statistically evaluated. According to their performance, a score was assigned to each model, and the models with the highest ranks were selected (Table 2).

**Table 2:** GCM performance for observed rainfall data.

GCMs	pbias	score	rmse (%)	score	r2	score	corr	score	Total	Rank
bcc_csm1_1	–44.9	20	10.41	24	–3.47	21	–0.186	21	86	25
bcc_csm1_1_m	–26.8	8	8.28	20	–5.7	24	0.116	4	56	15
bnu_esm	–35.1	11	8.79	22	–0.98	3	0.231	2	38	4
cccma_canesm2	–12.9	2	6.75	8	–3.23	20	–0.098	15	45	6
cesm1_cam5	–17.8	5	5.68	4	–1.36	6	0.038	7	22	1
cnrm_cm5	–39.5	15	10.46	26	–2.15	12	0.176	3	56	16
csiro_access1_0	–57	23	5.12	2	–1.81	8	–0.194	23	56	17
csiro_mk3_6_0	–43.4	19	7.29	13	–3.1	19	–0.131	19	70	22
gfdl_cm3	–43.3	18	6.97	10	–2.93	17	–0.044	9	54	14
gfdl_esm2g	–36.2	12	4.81	1	–3.03	18	–0.188	22	53	12
gfdl_esm2m	–33.8	10	7.97	17	–2.14	11	–0.047	10	48	9
inm_cm4	–13.2	3	6.68	7	–4.56	23	–0.179	20	53	13
ipsl_cm5a_lr	–49	21	10.45	25	–1.13	5	–0.285	26	77	24
ipsl_cm5a_mr	–41.2	17	7.14	11	–3.77	22	–0.061	11	61	21
ipsl_cm5b_lr	–66.1	26	7.14	12	–0.43	1	0.098	6	45	7
lasg_fgoals_g2	–30.5	9	7.69	15	–5.76	26	–0.196	24	74	23
miroc_esm	–37.6	13	8.15	19	–2.28	13	–0.095	13	58	19
miroc_esm_chem	–38.3	14	7.43	14	–2.41	14	–0.111	17	59	20
miroc_miroc5	–19.8	6	7.71	16	–1.84	9	–0.201	25	56	18
mohc_hadgem2_cc	–63.2	24	8.51	21	–5.74	25	–0.1	16	86	26
mohc_hadgem2_es	–65	25	5.87	5	–0.99	4	–0.098	14	48	10
mpi_esm_lr	–9.1	1	8.08	18	–1.99	10	–0.124	18	47	8
mpi_esm_mr	–17.7	4	6.18	6	–2.67	16	0.099	5	31	2
mri_cgcm3	–52.3	22	5.66	3	–1.76	7	–0.089	12	44	5
ncar_ccsm4	–19.9	7	9.49	23	–0.61	2	0.242	1	33	3
ncc_noresm1_m	–40	16	6.79	9	–2.66	15	–0.039	8	48	11

Based on the analysis of the observed climate data along with the GCM data, four GCMs, namely, mpi-esm-mr developed by the Max Planck Institute for Meteorology, Germany, bnu-esm developed by the Beijing Normal University, China, and cesm1-cam5 and ncar-ccsm4 developed by the National Center for Atmospheric Research, USA, were used to simulate the reasonable present-day climatology and to study the future climate scenarios.

### Intensity-duration-frequency curves

In order to reiterate the relationship between the precipitation dynamics that is used to deal with the catchment hydrology, IDF curves were used [38]. The IDF curves for different durations were generated with an annual maximum series of rainfall data by using the frequency analysis [39]. In this study, the IDF curves were developed for five different return periods of 2, 5, 10, 50, and 100 years. For the development of the IDF curves, the daily rainfall data of the past and future climate scenarios were recorded. From the daily rainfall data, the hourly rainfall data were calculated by using the following empirical reduction formula of IMD [40]:

$$P_{t}=P_{24}\sqrt[{3}]{\frac{t}{24}}$$ (1)

where P_t_—Required rainfall depth in mm at t–h duration,

P_24_- Daily rainfall in mm,

t—Duration of rainfall in hour.

The hourly rainfall values for different durations, such as 1 h, 2 h, 6 h, 12 h, and 24 h, were calculated from the annual maximum values. For different durations, the mean and standard deviation of the data were calculated and presented in Table 3.

**Table 3:** Hourly rainfall at Meenambakkam with mean and standard deviation.

Duration	1 h	2 h	6 h	12 h	24 h
**Mean (mm/h)**	54.77	34.50	16.59	10.45	6.58
**Standard deviation (mm/h)**	24.99	15.75	7.57	4.77	3.00

Gumbel’s extreme value distribution is most commonly used for IDF relationships and in this study, it is used to fit the probability distribution [16]. For different return periods, the *K*_T_ values were calculated by using Gumbel’s distribution. Moreover, for a given return period, the rainfall intensity was calculated by using the frequency factors as shown by the following equation:

$$x_{T}=\overset{˘}{x}+K_{T}s$$ (2)

where *x_T_*—Rainfall intensity at given return period,

$\overset{˘}{x}$—Mean of the particular time,

s—Standard deviation,

K_T_—Frequency factor.

Based on the observed rainfall data (1975–2015) and the projected rainfall data from the four GCMs (2015–2085), the IDF curves were generated for different return periods, namely, 2, 5, 10, 50, and 100 years, respectively.

### Base map preparation and land use classification

The study area boundary that covers the entire Adyar sub-basin and its surrounding areas was digitized by using toposheets 66 D 1&5, C4, C8, 57 P 13, and 57 O 16 collected from the Survey of India (SoI) and the administrative map as shown at www.cmdamaps.nic.in. For the year 2016, the Landsat TM images of 30 m resolution were downloaded from http://landsatlook.usgs.gov/viewer.html. By using the topographic maps of the Adyar sub-basin at the 1:50000 scale, a geometric correction was performed on the satellite image. First, the satellite image was geo-referenced in the cartographic projection (UTM Zone 44N, WGS84). Then, the satellite image was clipped along with the boundary of the study area. The satellite image was digitized manually by using the ArcGIS software in order to analyze and compute the areas under each land use class as adopted in the LULC scheme. Considering the standard Level I classification defined in an earlier report [41] and the local factors such as topography, land use, etc., five separate LULC classes, namely, agriculture land, barren land, forest cover, settlements, and water bodies, were defined. By using the Google Earth Explorer, the accuracy of the satellite image was cross-verified.

### Digital elevation model

High-resolution Digital Elevation Model (DEM) data are considered to be extremely useful in establishing the hydrologic and hydraulic models in order to provide accurate flood depth and extent [42]. The CartoDEM data of 10 m resolution were obtained by the National Remote Sensing Centre (NRSC), Hyderabad, India. The CartoDEM is a surface model of elevation, which covers the entire land surface of India. It is constituted of tiles with a size of 13.5 km × 13.5 km [43]. The whole study area was covered by the following tile numbers: D44U–1-NW, D44U–1-NE, D44U–1-SW, D44U–1-SE, D44U–1-NW, D44U–1-SW, D44T–13-NW, D44T–13-NE, D44T–13-SW, D44T–13-SE, D44O–4-SE, D44O–4-SW, D44O–8-SW, D44N–16-SE, and D44N–16-SW. These 15 tiles were supplied by the NRSC. Moreover, all the procured tiles were merged and geo-corrected in ERDAS IMAGINE 2014. In order to delineate the flow direction and the stream network in the Adyar sub-basin, the high-resolution CartoDEM data were used.

### Rainfall-runoff modeling using HEC-HMS

**Fig 2:**
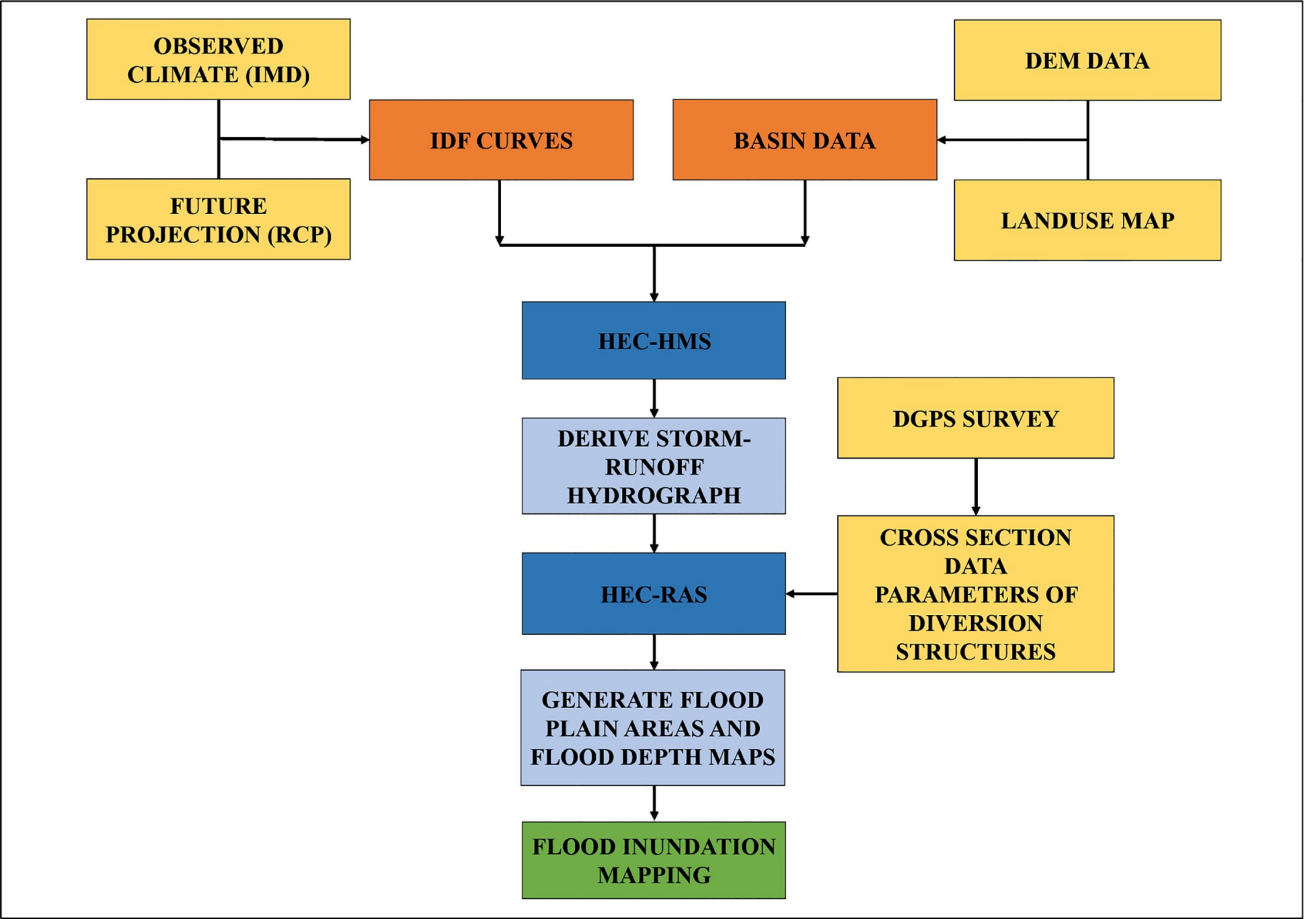
Methodology framework adopted in this study.

In order to assess the fluvial flooding according to the present and future climate change scenarios, the integrated hydrologic and hydraulic modeling was used in this study. The methodological framework followed in this study is presented in Fig 2.

**Fig 3:**
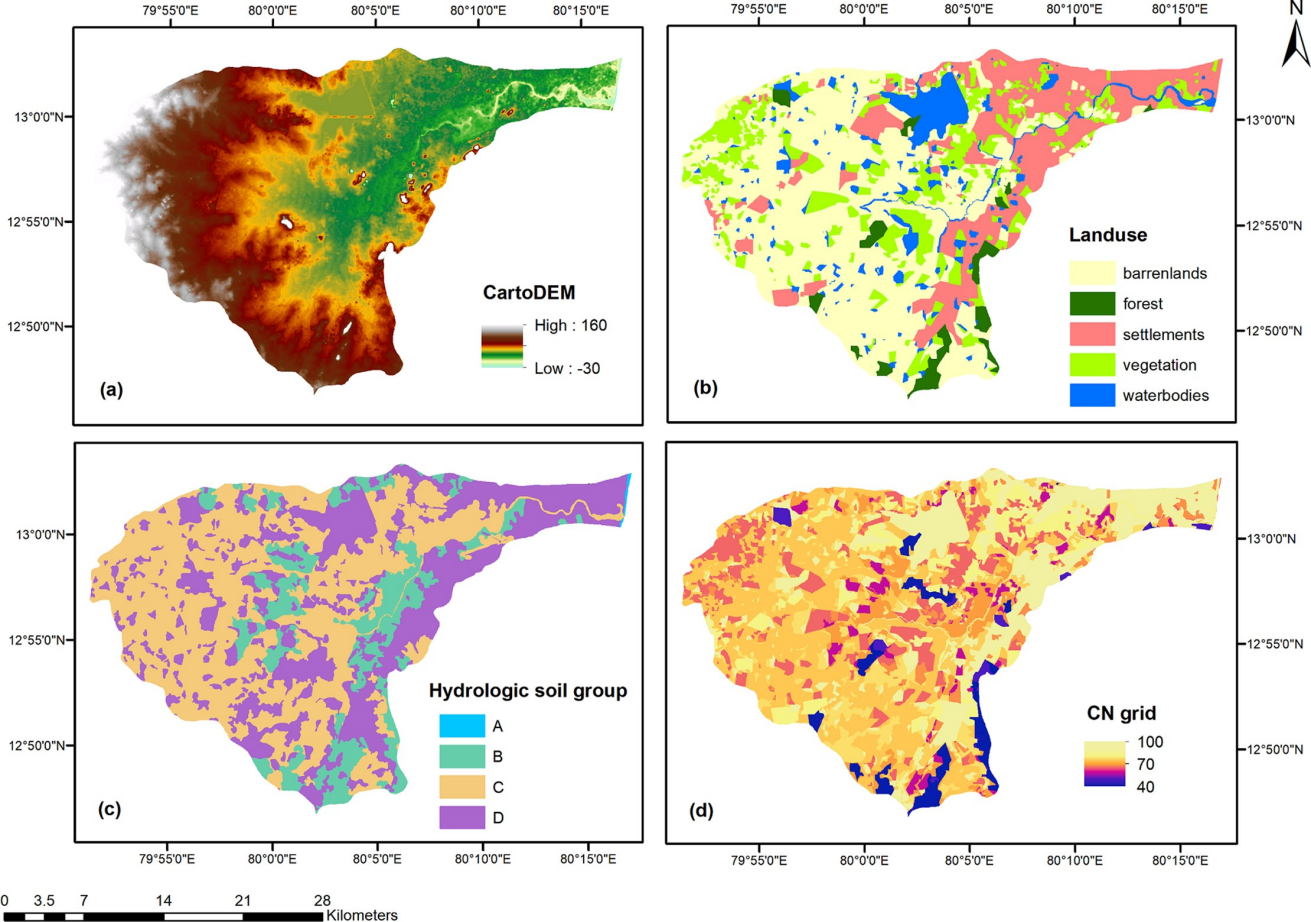
Characteristics of the Adyar Sub-basin. (A) Digital elevation model, (B) Land use, (C) Hydrologic soil group, (D) Curve number grid.

In order to simulate the rainfall-runoff processes of a river basin, the Hydrologic Engineering Center-Hydrologic Modeling System (HEC-HMS) developed by the U.S. Army Corps of Engineer is widely used. The basin model, meteorological model, and control specifications are the main components of HEC-HMS [44]. The basin model provides the physical representation of the sub-basin which was developed by using the ArcGIS Software. The Arc Hydro tool, an extension in ArcGIS, was used to process the DEM data, to delineate the sub-basin and the stream network, and to extract the sub-basin properties, such as area, slope, flow length, stream network density, etc. Moreover, land use information, hydrologic soil group, and curve number were used as the inputs to the model. Based on the soil data collected from the Institute of Remote Sensing, Anna University, the hydrologic soil group map of the study area was prepared. Furthermore, the weighted curve number (CN) map was generated for the sub-basin considering the land use data, hydrologic soil group, and curve numbers. Digital elevation model, Land use, Hydrologic soil group and Curve number grid of the Adyar sub-basin are shown in Fig 3A, Fig 3B, Fig 3C and Fig 3D respectively.

The SCS method is considered to be a simple and stable method. Generally, it is applied in different environments and provides good results [45]. The SCS method was used for model simulation, whereas the Muskingum method was used for channel routing. The SCS unit hydrograph (UH) was used for sub-basin transformation. By assuming the time of concentration as the duration of rainfall in a basin, the critical rainfall depth for a selected return period is determined by using the IDF curves. This critical rainfall depth is distributed over time across the basin [25]. This rainfall intensity was considered as an input to the meteorological model of the HEC-HMS. The start and end of the computation period and time interval were specified in the control specifications. According to the present and future climate scenarios, the simulation run for the Adyar sub-basin was processed and the runoff hydrograph was generated for different return periods. In order to calculate flood inundation, these hydrographs were used as inputs for the HEC-RAS model.

### Hydraulic modeling using HEC-RAS

HEC-RAS is a one-dimensional hydraulic model, which is used to simulate the surface water elevation along the river for the specified discharge hydrographs. The HEC-RAS model requires geometric data and flow data for simulation [46]. HEC-GeoRAS is an extension of ArcGIS, which is used to create geometric data consisting of the river centerline, bank lines, flow path lines, and cross-sectional details. In order to extract the profiles of river station and elevation data along the cross-sections, the CartoDEM of 10 m resolution was used. By using the Leica DGPS instrument, a field survey was carried out to measure the elevation of the points along the banks of the Adyar river. An Acoustic Doppler Current Profiler (ADCP) (Qliner) instrument was used to measure the elevation of the river bed. The geographical co-ordinates of the field survey points are shown in Table 4.

**Table 4:** Geographical location of field survey points.

ID	LATITUDE	LONGITUDE
1	12°55'17.94"N	80° 4'35.36"E
2	12°57'56.15"N	80° 6'37.82"E
3	12°59'8.14"N	80° 7'14.65"E
4	13° 1'7.26"N	80°11'9.72"E
5	13° 1'35.87"N	80°12'26.31"E
6	13° 1'0.00"N	80°13'0.44"E
7	13° 1'6.95"N	80°13'29.16"E
8	13° 1'31.52"N	80°14'37.26"E
9	13° 0'39.79"N	80°15'31.67"E

No specific permissions were required for these locations where DGPS survey and Qliner survey was carried out since the survey was not carried out in any private ownership land or protected area of land. This field survey did not involve any endangered species or protected species. To cross-verify the geometric data extracted from the DEM data, the above survey points were used. The details of bridges, such as a number of piers, pier width, roadway width, etc., were also collected from the field survey. These geometric data were then exported by using the HEC-RAS model. The steady flow data were used as inputs in the HEC-RAS model. The steady flow data consisted of flow type and boundary conditions. In this study, the flow type was assumed as mixed flow and the required boundary conditions were applied in the upstream and downstream ends of the river. The hydrographs derived from the HEC-HMS model were stored as the data storage system (DSS) format that can be opened in the HEC-RAS model [46]. The peak discharge data were derived from the hydrographs and assigned to the corresponding junctions in the HEC-RAS model.

The HEC-RAS model simulated the water surface profile across all the cross-sections of the river. The flood depths for all cross-sections were computed as the peak discharges for the 2, 5, 10, 50, and 100-year return periods were calculated for the observed conditions. For the future projection of rainfall, the hydrographs generated from HEC-HMS for the selected four projected GCMs, namely, ncar-ccsm4, mpi-esm-mr, cesm1-cam5, and bnu-esm, were considered as flow inputs for the HEC-RAS model. The HEC-RAS model simulated the water surface profile for the projected GCMs and the flood depths were calculated. For the observed and projected scenarios, floodplain polygons were created for all return periods.

### Flood inundation mapping using ArcGIS

In order to understand the effects of floods from a particular river, flood inundation maps are required. Both the water surface profile and floodplain extent are involved in flood inundation mapping [8]. The water surface profile and floodplain polygon that were simulated using HEC-RAS were postprocessed by using the HEC-GeoRAS extension in ArcGIS. The water surface profile consisted of water surface elevation at a point over the entire sub-basin. The difference between the water surface elevation and the terrain elevation from the DEM data provided the flood depth at a point in the sub-basin. The flood depth data were overlaid on the satellite image in ArcGIS. Based on the flood depths in the range of 0–1 m, 1–2 m, 2–4 m, 4–6 m, and >6 m, the flood inundation maps were prepared for the observed and projected climate change scenarios with different return periods.

## Results and discussion

### Analysis of the past and projected rainfall

**Fig 4:**
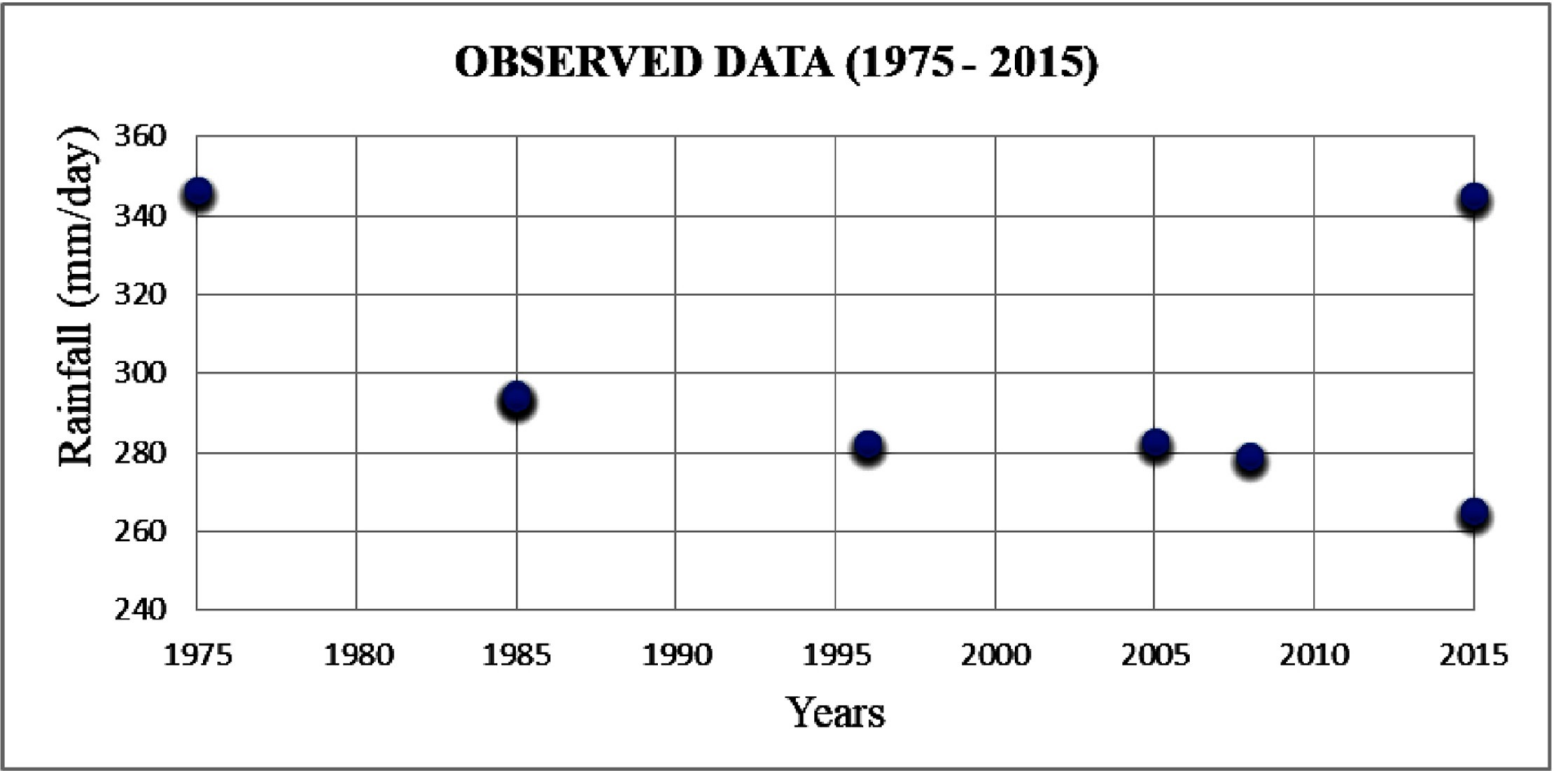
Extremely heavy rainfall events based on the observed data (1975–2015).

For the period 1975–2015, the average annual rainfall of the Meenambakkam station was observed to be 1472 mm. The post monsoon (October to December) rainfall was the major contributor (59.6%) of the annual rainfall and it received an average rainfall of 878 mm. The average rainfall in the monsoon season (June to September) was 479 mm and the monsoon contribution to the annual rainfall was about 32.6%. The summer (March to May) rainfall contributed 4.9% and the average rainfall was 72.9 mm. The average rainfall during winter (January to February) was 41.8 mm and the winter rainfall contributed 2.8% of the annual rainfall. For the observed rainfall data, the extremely heavy rainfall events (>244.5 mm/day) were analyzed according to the IMD standards during the period 1975–2015 (Fig 4).

**Fig 5:**
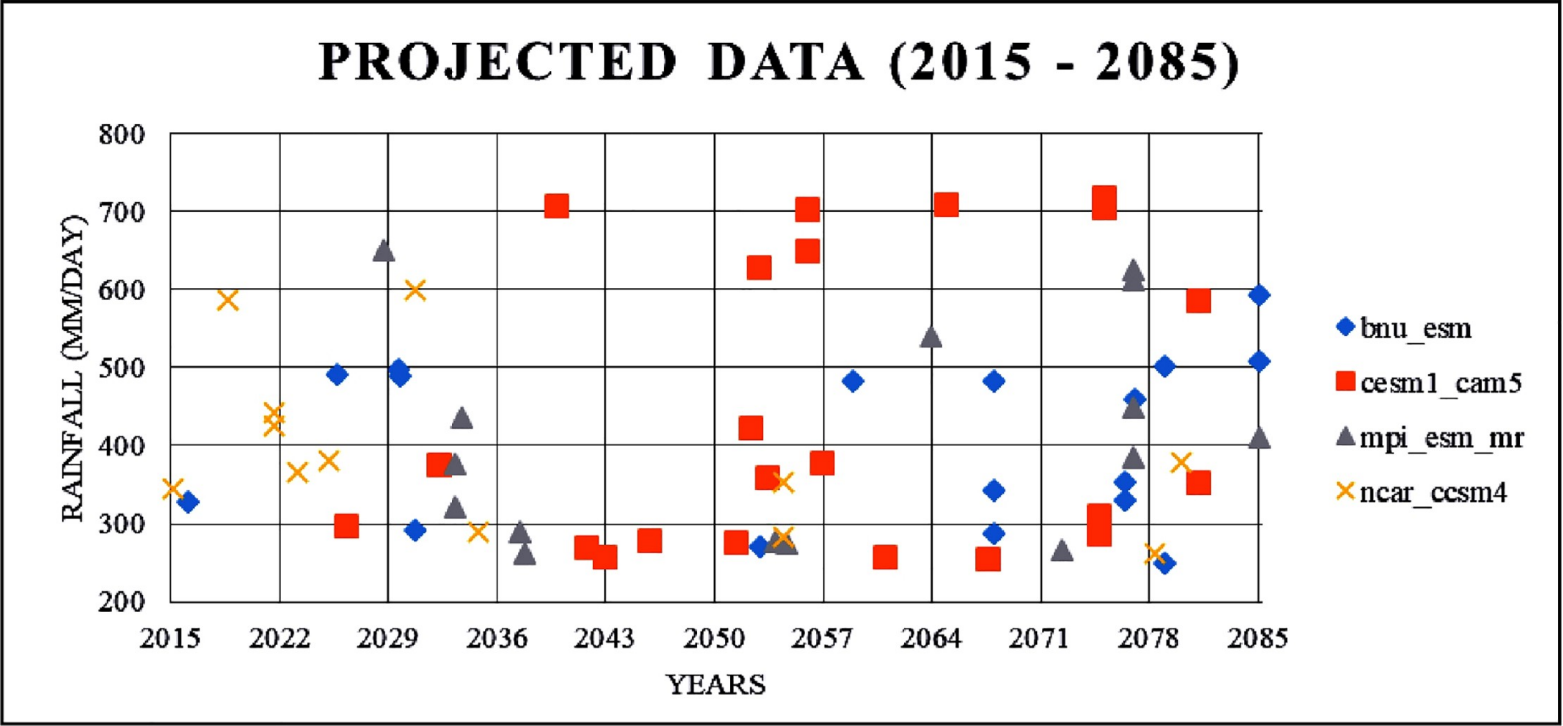
Extremely heavy rainfall events based on the projected data (2015–2085).

During the past three decades, eight extremely heavy rainfall events were recorded. During 2005–2015, four extremely heavy rainfall events were recorded and two of these events occurred in the year 2015 (November to December). During 2015–2085, extremely heavy rainfall events were also analyzed for the GCM future projection data (Fig 5).

The projected rainfall pattern shows that the number of extremely heavy rainfall events will be increasing after the mid-century, i.e., 2050. Evidently, the projected rainfall intensity becomes nearly double that of the observed rainfall intensity.

### Intensity-duration-frequency curves

**Fig 6:**
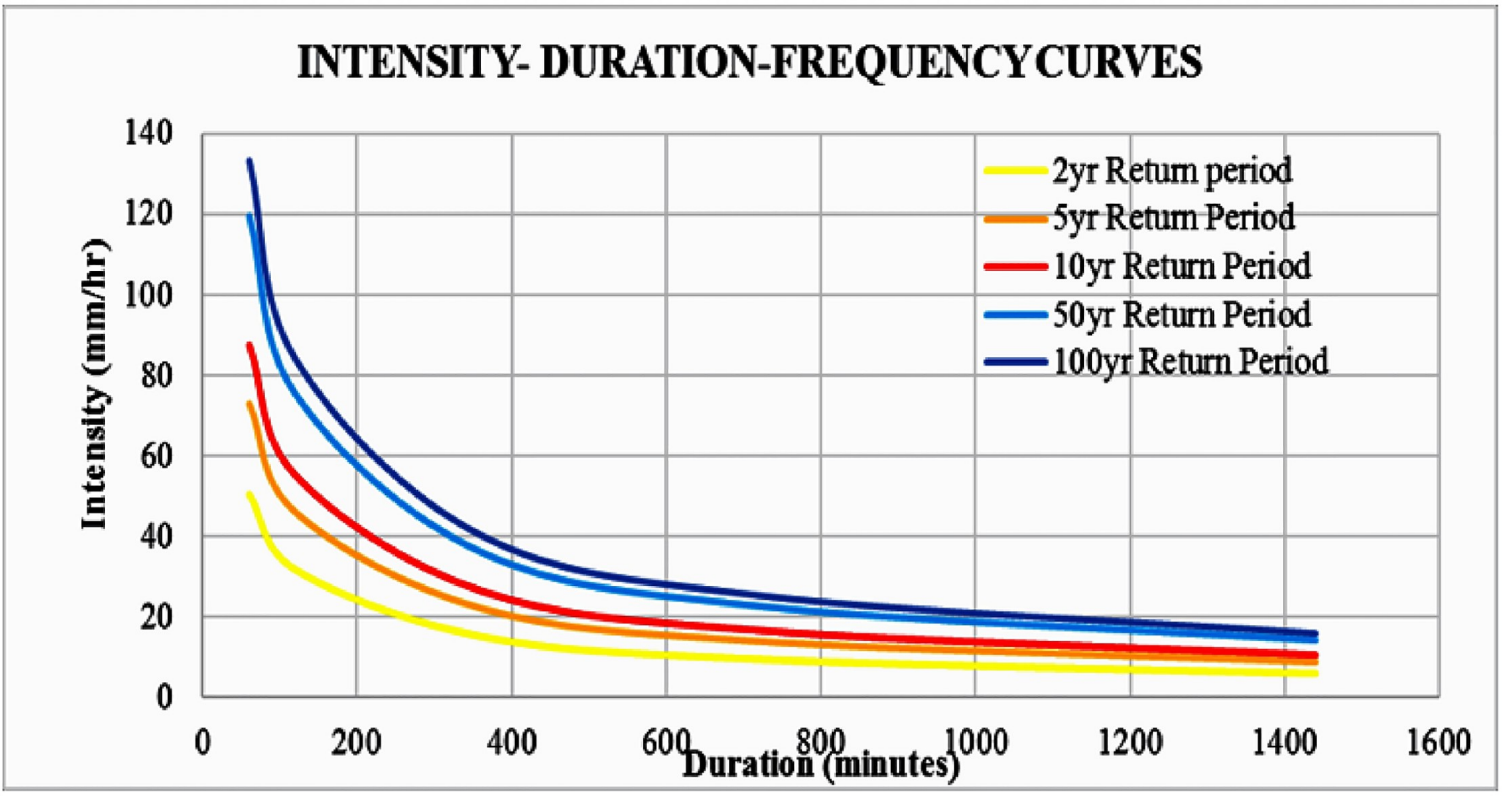
IDF curves for Meenambakkam station rainfall for the period 1975–2015.

For the period 1975–2015, the IDF curves were developed for five return periods of 2, 5, 10, 50, and 100 years based on the observed rainfall data from the Meenambakkam station (Fig 6).

**Fig 7:**
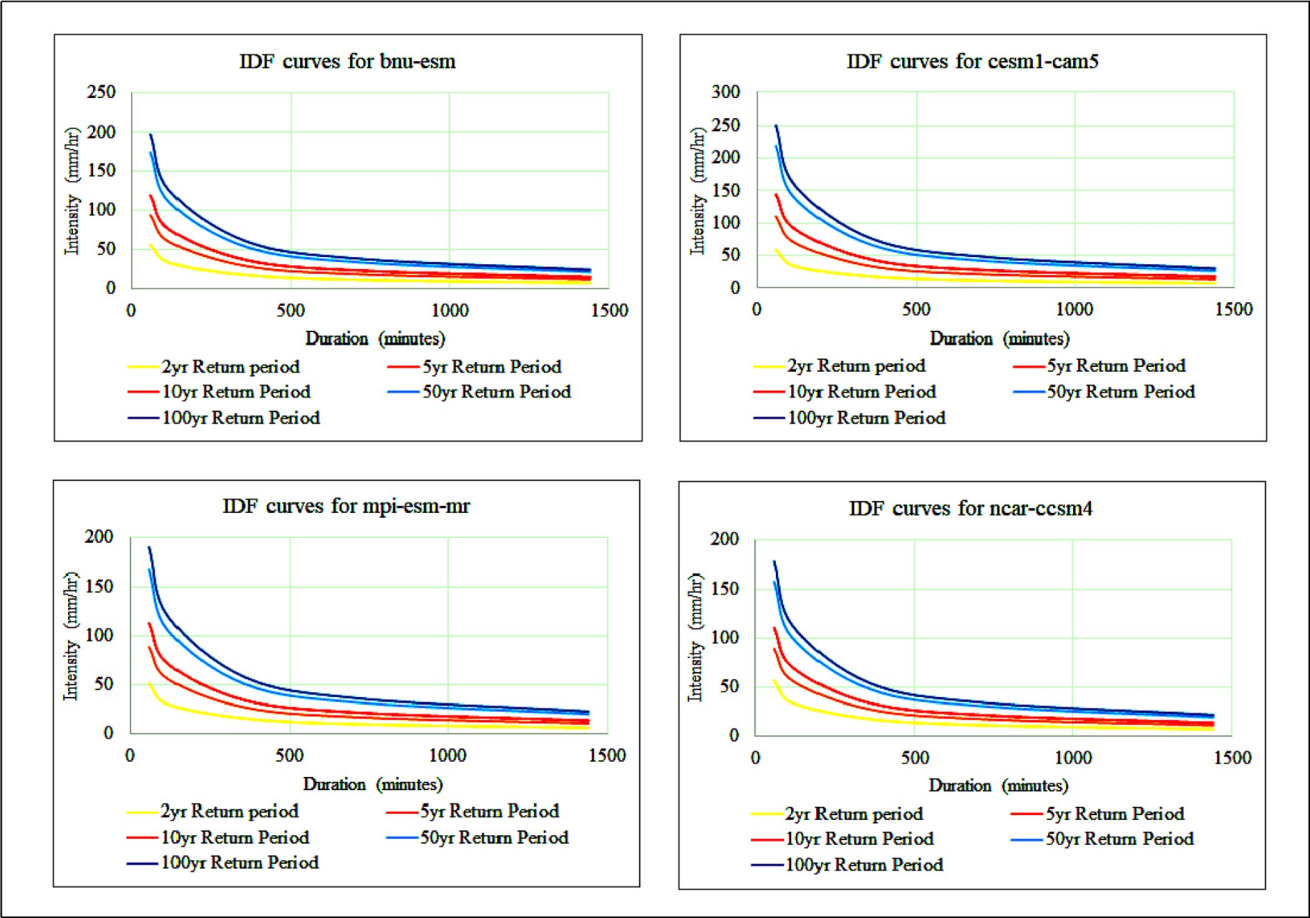
IDF curves for four GCMs used in this study for the period 2015–2085.

IDF curves were also developed for the selected GCMs, namely, cesm1-cam5, mpi-esm-mr, ncar-ccsm4, and bnu-esm, under the RCP 4.5 scenario for the period 2015–2085 (Fig 7). Based on the time of concentration, the rainfall intensities were calculated from the IDF curves for all return periods.

### Land use

**Fig 8:**
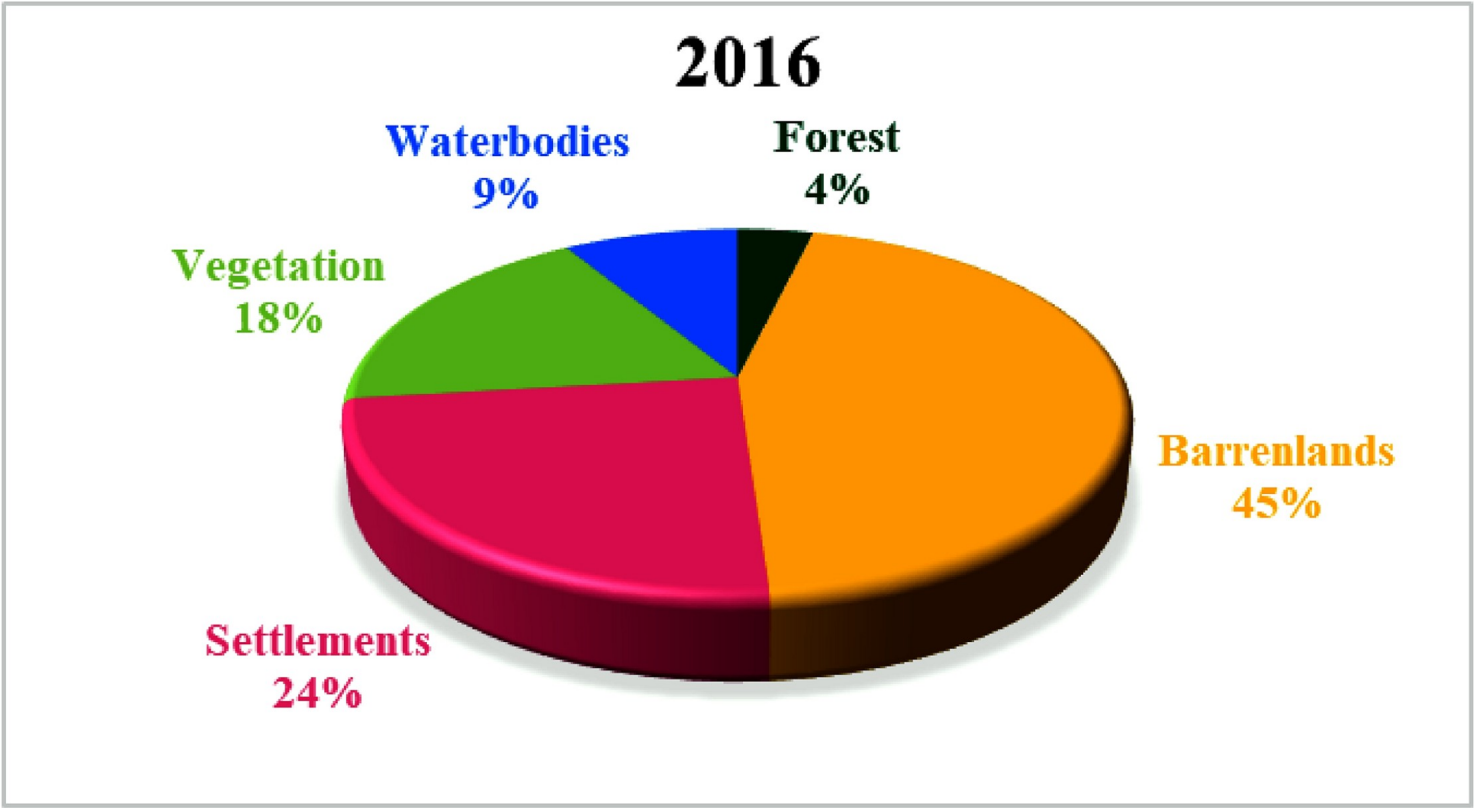
Land use pattern for the year 2016.

The land use map was prepared using the Landsat-8 satellite image for the year 2016. The land use of the Adyar sub-basin was divided into five classes, namely, barren lands, settlements, vegetation, water bodies, and forest. Fig 3B depicts the land use map of the Adyar sub-basin. The total area of the Adyar sub-basin is about 700 km^2^, and 317 km^2^ of this area is barren land that covers almost half of the sub-basin. Settlements cover nearly one-fourth of the total sub-basin area, which is around 170 km^2^. Vegetation covers 18% of the total sub-basin area, which is around 127 km^2^. Water bodies cover 9% of the total sub-basin area, which is around 61 km^2^. Forest covers around 26.3 km^2^, which is 4% of the total sub-basin area. Fig 8 shows the land use covered by each category for the year 2016. The land use map was used in hydrological modeling.

### Hydrologic modeling

The hydrologic model HEC-HMS was used to simulate flood hydrographs over the Adyar sub-basin. The model simulated a hydrograph with a peak discharge of 3141 m^3^/s for December 2015 rainfall event. Since there was no observation of that particular event, experts suggested that a peak discharge was approximately above 2800 m^3^/s [47,48]. Evidently, there was a fair agreement between model simulation and the opinion of experts.

**Fig 9:**
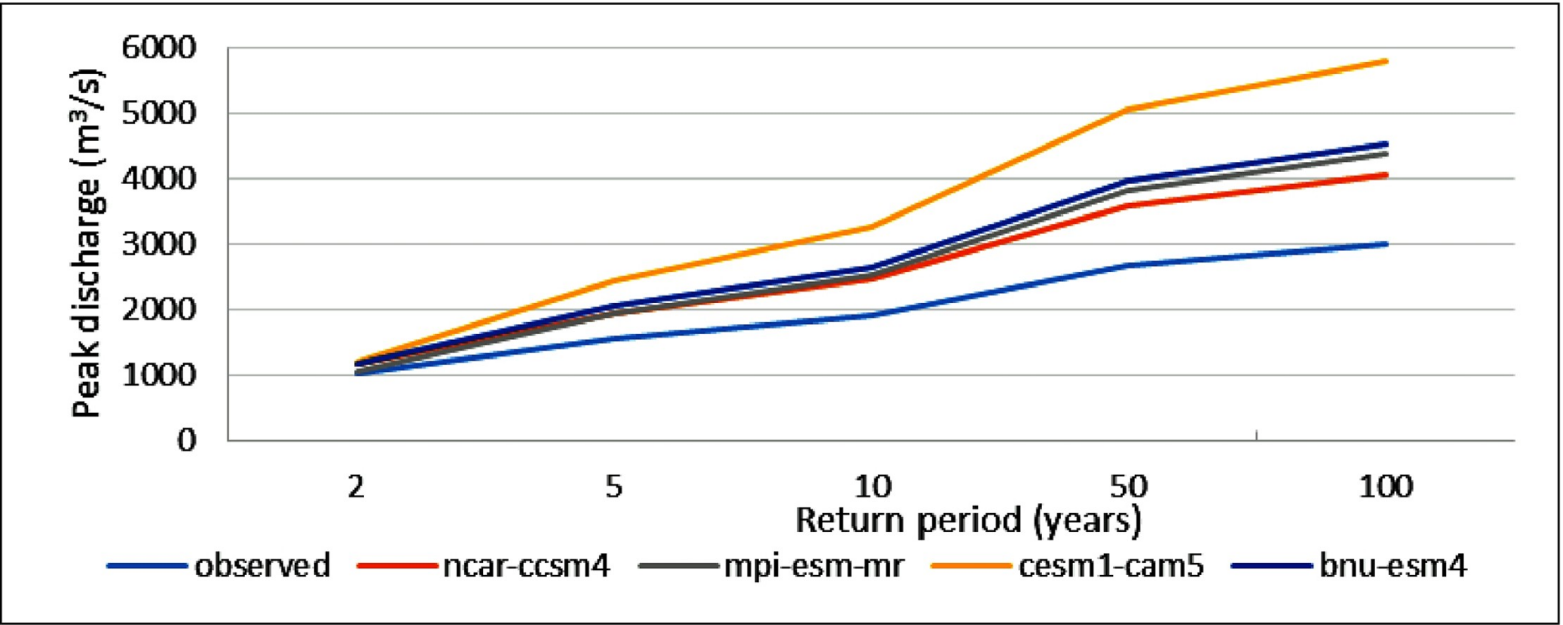
Comparison of peak discharge between observed and projected GCMs for different return periods.

The present flood discharge capacity of the Adyar river is around 1200 m^3^/s, whereas the expected flood discharge capacity is about 1800 m^3^/s. (http://www.cmdachennai.gov.in/pdfs/SeminarOnWaterways/5.pdf). Based on the observed data, the model simulated the peak discharge of 1021 m^3^/s, 1548 m^3^/s, 1896 m^3/^s, 2665 m^3^/s, and 3022 m^3^/s for the 2, 5, 10, 50, and 100-year return periods, respectively. It is clear from the model simulation results that flood events might occur for the 10-year return period and above because the peak discharge exceeds the flood-carrying capacity of the Adyar river. For the period 2015–2085, peak discharges of the Adyar river for different return periods were simulated according to the future climate scenarios. For the 2-year return period, the peak discharge ranges from 1054 m^3^/s to 1190 m^3^/s. For the 100-year return period, the peak discharge ranges from 4060 m^3^/s to 5800 m^3^/s, which is almost double that of the past climate scenarios. A comparison of the peak discharges shows a significant increase in the observed and projected GCMs for the 2, 5, 10, 50, and 100-year return periods (Fig 9).

### Hydraulic modeling

The HEC-RAS model is used to simulate the flood depths and flood extent. The flood inundation depth was calculated by intersecting the water surface elevations with the DEM data. Based on the observed data, the flood depths and flood inundation areas were calculated for the 2, 5, 10, 50, and 100-year return periods. For different return periods, the flooding pattern varies according to the present scenario as shown in Table 5.

**Table 5:** Flooding pattern of Adyar basin for return periods.

Return Period (years)	Flooded area (km^2^)	% increase in flooded area
2	33.02	
5	39.38	19.3
10	44.21	33.9
50	49.64	50.3
100	52.14	57.9

**Fig 10:**
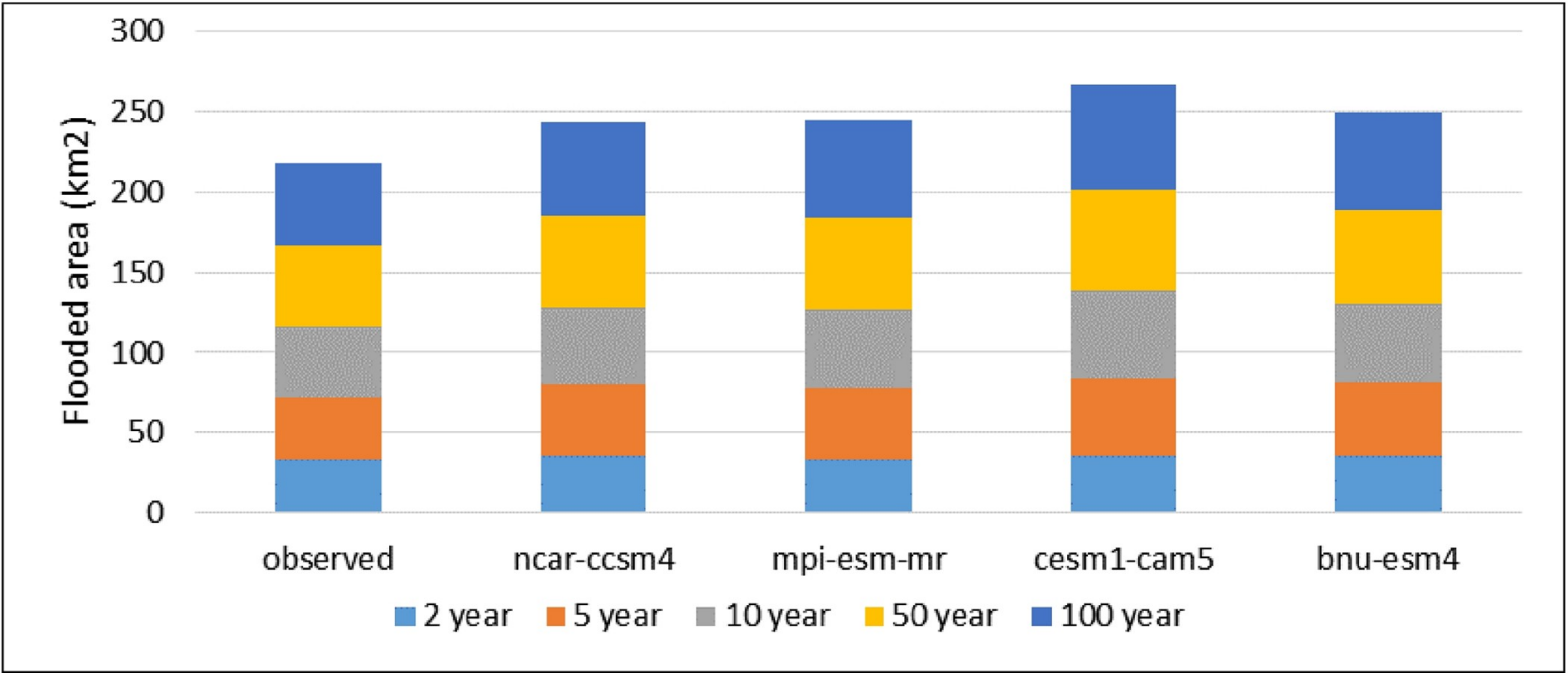
Comparison of the flooded area between observed and projected GCMs for different return periods.

For a normal 2-year storm event, nearly 32 km^2^ of the area would be inundated in the Adyar basin. However, for a 5-year storm event, 20% increase is observed in the flooded area, for a 10-year return period, around 31% increase is observed in the flooded area, and for a 50-year storm event, 50% of increase is observed in the inundated area. However, for the 100-year event, 59% increase is observed in the inundated area. Similarly, the flooding pattern was calculated for different return periods according to future conditions. For different return periods, a comparison of the flood area between the observed and projected GCMs is shown in Fig 10. It shows a significant change in the projected flooded area and the observed data. For the 2-year return period, no significant increase is reported in the flooded area between the observed and projected GCMs. For the 100-year return period, the flooded area of 58.69 km^2^, 60.06 km^2^, 65.89 km^2^, and 61.16 km^2^ was observed for ncar-ccsm4, mpi-esm-mr, cesm1-cam5, and bnu-esm4, respectively. Evidently, it is reported that the projected flooded areas have significantly increased as compared to those of the flooded areas under the observed conditions.

### Flood mapping

**Fig 11:**
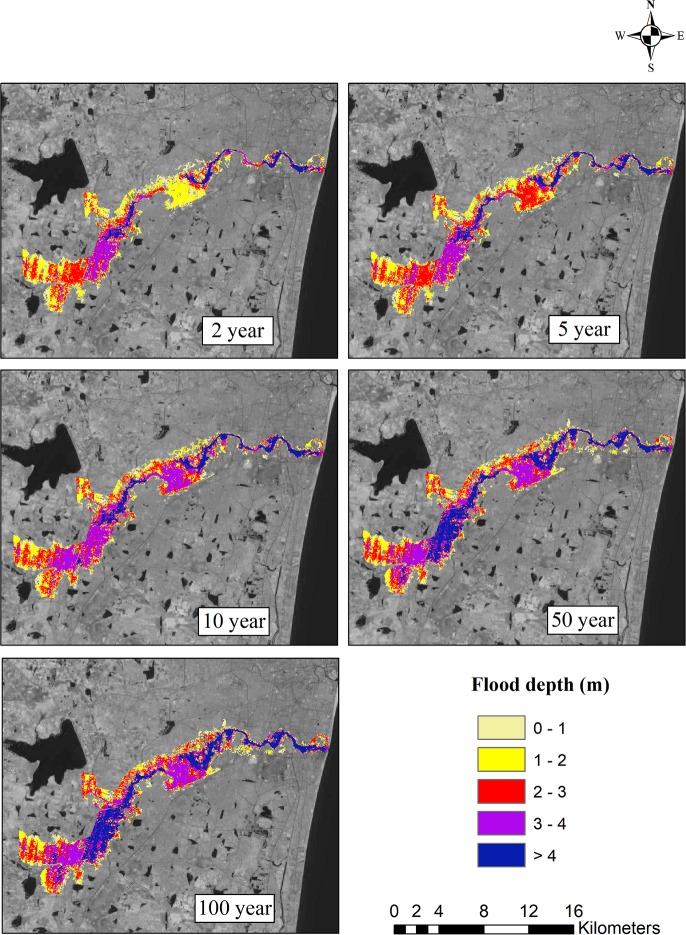
Flood inundation map for 2, 5, 10, 50 and 100 year rainfall return period based on observed data (1975–2015). Landsat image used as a base map in this figure, was obtained from the USGS EROS (Earth Resources Observatory and Science (EROS) Center) (public domain): http://eros.usgs.gov/#.

**Fig 12:**
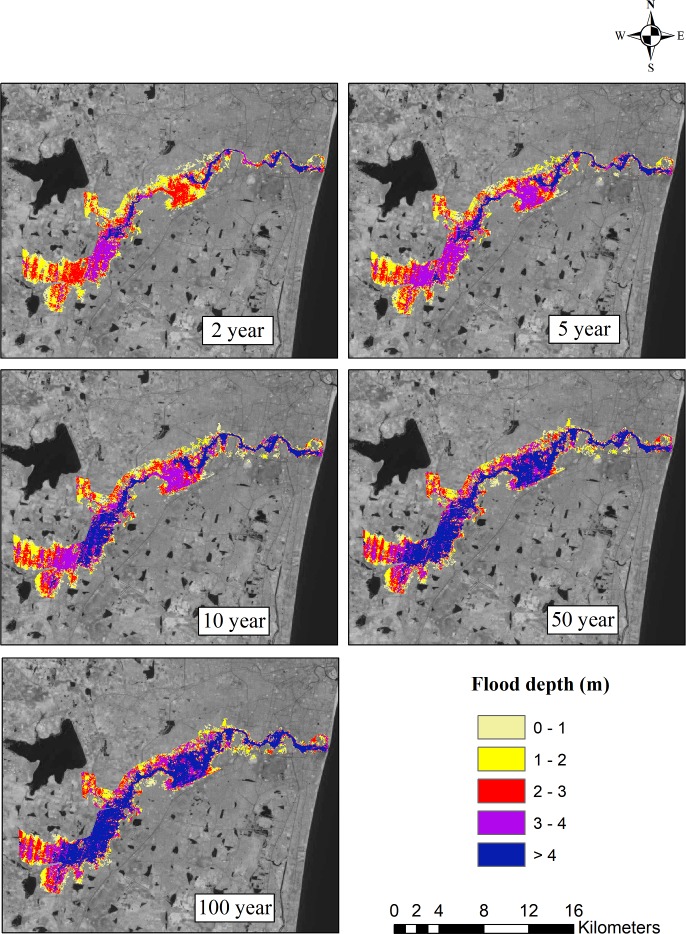
Flood inundation map for 2, 5, 10, 50 and 100 year rainfall return period based on ncar-ccsm4 model (2015–2085). Landsat image used as a base map in this figure, was obtained from the USGS EROS (Earth Resources Observatory and Science (EROS) Center) (public domain): http://eros.usgs.gov/#.

**Fig 13:**
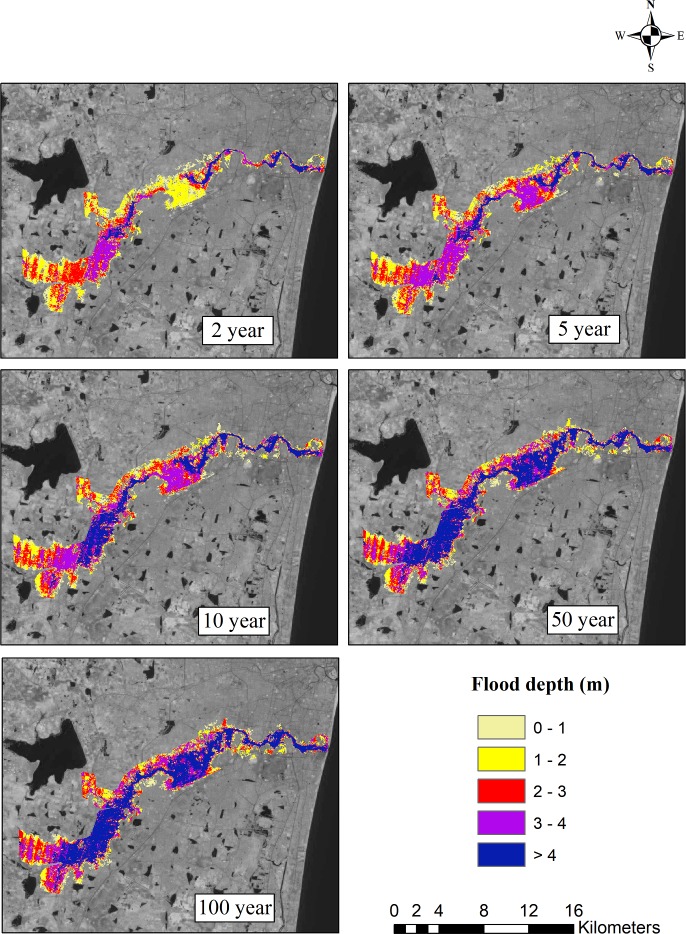
Flood inundation map for 2, 5, 10, 50 and 100 year rainfall return period based on mpi-esm-mr model (2015–2085). Landsat image used as a base map in this figure, was obtained from the USGS EROS (Earth Resources Observatory and Science (EROS) Center) (public domain): http://eros.usgs.gov/#.

**Fig 14:**
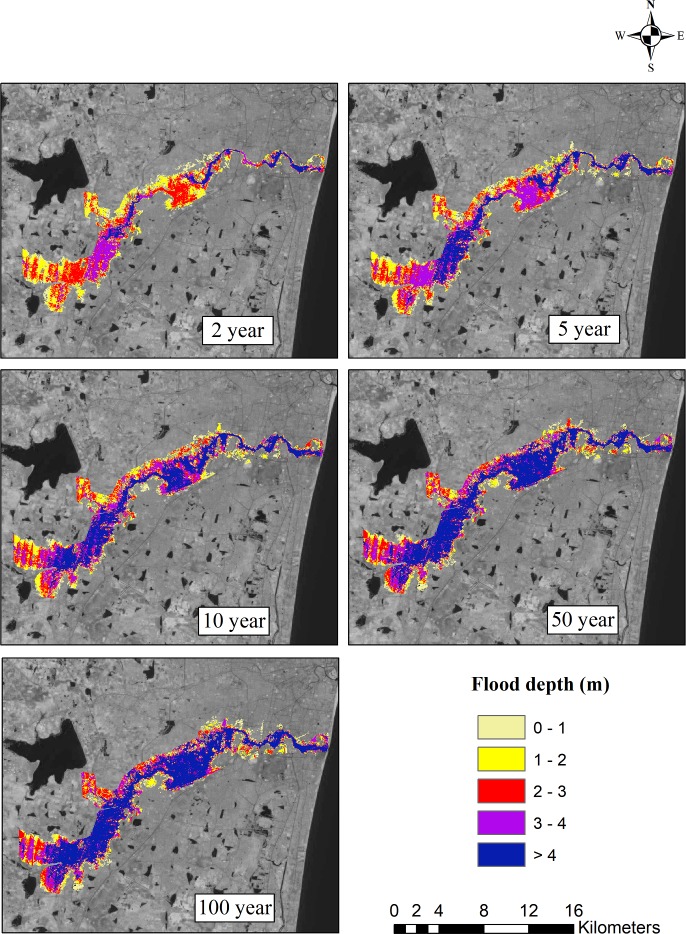
Flood inundation map for 2, 5, 10, 50 and 100 year rainfall return period based on cesm1-cam5 model (2015–2085). Landsat image used as a base map in this figure, was obtained from the USGS EROS (Earth Resources Observatory and Science (EROS) Center) (public domain): http://eros.usgs.gov/#.

**Fig 15:**
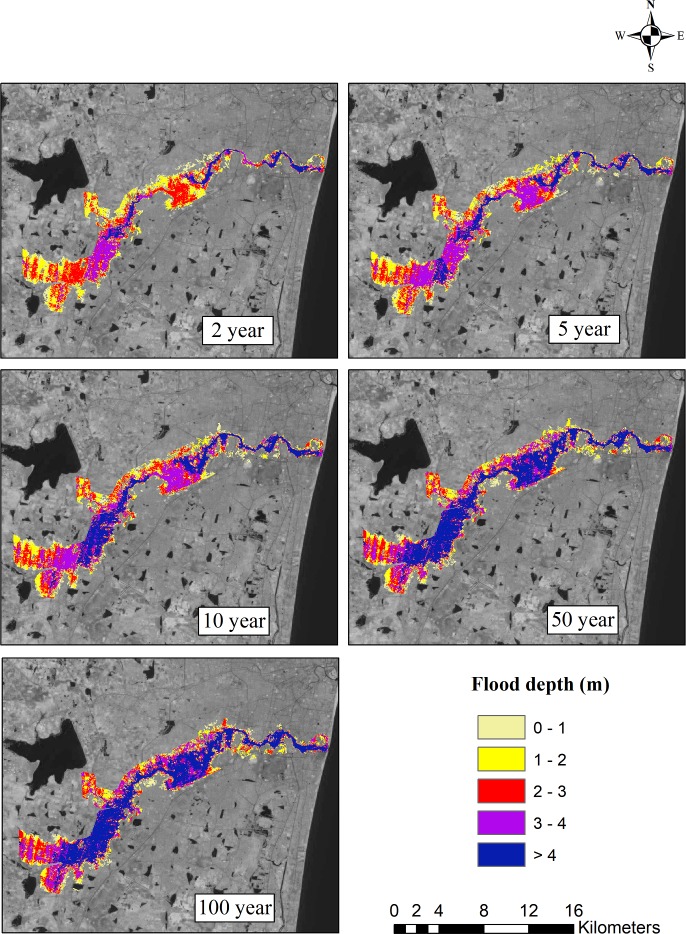
Flood inundation map for 2, 5, 10, 50 and 100 year rainfall return period based on bnu-esm4 model (2015–2085). Landsat image used as a base map in this figure, was obtained from the USGS EROS (Earth Resources Observatory and Science (EROS) Center) (public domain): http://eros.usgs.gov/#.

The ArcGIS software was used to prepare flood inundation maps. In order to understand the significant changes in the flooded area according to the past and future climate change scenarios, these flood inundation maps were further analyzed. Based on the observed data, the flood inundation map for the 2, 5, 10, 50, and 100-year return periods is shown in Fig 11. For different return periods, flood inundation maps were also generated for all four GCMs. The flood inundation maps for the projected GCMs ncar-ccsm4, mpi-esm-mr, cesm1-cam5, and bnu-esm4 for the 2, 5, 10, 50, and 100-year return periods are shown in Figs 12–15. The flood inundation maps show that there is an increase in the flood depth from 1 m to 4 m around the Adyar river. It is observed that the upstream end of the river is more vulnerable as compared to the downstream end of the river. Based on the future projections, it is found that areas such as Varadharajapuram, Mudichur, Ankaputhur, and Thirumudivakkam located in the upstream end of the river are more vulnerable to flooding. Moreover, it is also reported that areas such as Chennai International Airport, Ramapuram, Saidapet, and Kotturpuram located in the downstream end of the river are also vulnerable to flooding. Finally, it is concluded that these flood maps are helpful in identifying the flood-prone areas in the Adyar sub-basin.

## Conclusion and recommendations

This study is supported by 30 years (1975–2015) of the observed rainfall data collected from the IMD Meenambakkam station, 70 years (2016–2085) of the future projection data from the selected four GCMs, and GPS points collected from the field survey. The analysis of the past rainfall shows that there is an increase in the number of extreme events in the past decade. The rainfall projections also show that extreme events would be increasing in the future, which is a clear indication of the impact of climate change. An increase in the rainfall intensity leads to higher peak discharge and thus substantially increases the flood risks [49]. Hence, the projection of the rainfall intensity is considered important in all flood modeling studies. The IDF curves are considered useful for providing rainfall intensities for the area that receives high rainfall in a shorter duration. Based on the extreme rainfall intensity for the present and the future, the IDF curves are derived, which further help in identifying the extreme rainfall events that occur as a result of climate change.

This study successfully used the hydrologic and hydraulic models along with the GIS and also considered the climate change scenario for flood modeling over the Adyar sub-basin. Based on the model simulation, it is determined that the Adyar river can safely discharge the 2, 5, and 10-year floods. However, the 50 and 100-year floods are considered a threat for flooding over the Adyar river since its peak discharge exceeds the anticipated flood-carrying capacity. This fact is clearly justified by the past flood events that occurred in the Adyar river. According to the future scenarios, the increase in peak discharge ranges from 3.2% to 16.6% for the 2-year return period and from 34.3% to 91.9% for the 100-year return period. This is predicted by the 4 GCMs that were selected out of the 26 GCMs based on the higher ranked models in the statistical analysis. The cesm1-cam5 model was ranked 1st among the 26 GCMs, which estimated 91.9% increase in the peak discharge with 17.8% bias. The mpi-esm-mr model was ranked second, which estimated 44.3% increase in the peak discharge with 17.7% bias. The ncar-ccsm4 model was ranked third, which estimated 34.3% increase in the peak discharge with 19.9% bias. The bnu-esm model was ranked fourth, which estimated 49.9% increase in the peak discharge with 35.1% bias. This large fluctuation in the peak discharge is due to the presence of more extremities in the cesm1-cam5 model as compared to other GCMs. Analyzing the impact of extremities may be an additional approach in future studies. Moreover, different GCMs were used to project the impacts of climate change in this study but there exists some uncertainty associated with the GCMs, downscaling techniques and hydrological models [50]. Considering the changes in sea surface temperature (SST) patterns, internal variability, tropical cyclones etc., will be useful to quantify the uncertainties in the future projections which will be the future scope of the study[51,52]. The hydraulic model simulation shows that the water surface elevation exceeded the river banks in many cases. Hence, adequate measures are required to be taken in order to increase the flood discharge capacity of the river.

The flood inundation maps indicate that nearly 32 km^2^ area of Adyar is inundated for a normal 2-year storm event. However, a 20% increase in the flooded area is observed for the 5-year storm event, around a 31% increase in the flooded area is observed for the 10-year return period, nearly 50% increase in the inundated area is observed for the 50-year storm event, and a 60% increase in the area is observed for the 100-year storm event. Taking into consideration the future scenarios, the increase in the flooded area ranges from 1% to 7.8% for the 2-year return period and from 12.6% to 26.4% for the 100-year return period. The flood inundation maps depicts a clear picture about the impacts of climate change on the flooding pattern over the Adyar sub-basin. These results reveal that the peak discharge increases as a result of climate change, consequently increases the flood inundation over the Adyar sub-basin.

These observations clearly show that the increase in the flooded area will have some serious impacts on people living near the banks of the Adyar river. Furthermore, these flood inundation maps help in identifying the flood zones in the Adyar sub-basin, which can assist the government in decision making and policy framework. In addition, proper channelization of the Adyar river is required from the upstream end of the river. Similarly, the impact of flooding can also be decreased by providing adequate drains in the surrounding areas. Finally, it is recommended to construct the flood walls along the river bank in order to ensure the safety of the people. The above suggestions can certainly help in ensuring the safety of the people living near the Adyar river.
